# Immune mechanisms underlying COVID-19 pathology and post-acute sequelae of SARS-CoV-2 infection (PASC)

**DOI:** 10.7554/eLife.86014

**Published:** 2023-05-26

**Authors:** Sindhu Mohandas, Prasanna Jagannathan, Timothy J Henrich, Zaki A Sherif, Christian Bime, Erin Quinlan, Michael A Portman, Marila Gennaro, Jalees Rehman, RECOVER Mechanistic Pathways Task Force, Steven B Bradfute, Benjamin K Chen, Thomas J Connors, K Coombs, Christian R Gomez, Boris D Julg, C Kim, W Brian Reeves

**Affiliations:** Center for Global Health, Department of Internal Medicine, University of New Mexico Health Sciences CenterAlbuquerqueUnited States; Division of Infectious Diseases, Department of Medicine, Icahn School of Medicine at Mount SinaiNew YorkUnited States; Department of Pediatrics, Division of Critical Care, Columbia University Vagelos College of Physicians and Surgeons and New York - Presbyterian Morgan Stanley Children's HospitalNew YorkUnited States; NIH RECOVER Research Initiative: Patient representativeBethesdaUnited States; Division of Lung Diseases, National Institutes of Health, National Heart, Lung and Blood InstituteBethesdaUnited States; Infectious Disease Division, Massachusetts General Hospital, Ragon Institute of MGH, MIT and HarvardCambridgeUnited States; NIH RECOVER Research Initiative: Patient representativeBethesdaUnited States; Department of Medicine, Joe R. and Teresa Lozano Long School of Medicine, University of Texas San AntonioSan AntonioUnited States; 1 Division of Infectious Diseases, Children’s Hospital Los Angeles, Keck School of Medicine, University of Southern California Los Angeles United States; 2 Division of Infectious Diseases and Geographic Medicine, Department of Medicine, Stanford University Stanford United States; 3 Division of Experimental Medicine, University of California, San Francisco San Francisco United States; 4 Department of Biochemistry & Molecular Biology, Howard University College of Medicine Washington United States; 5 Division of Pulmonary, Allergy, Critical Care & Sleep Medicine, Department of Medicine, University of Arizona College of Medicine Tucson United States; 6 National Center for Complementary and Integrative Health, National Institutes of Health Bethesda United States; 7 Seattle Children’s Hospital, Division of Pediatric Cardiology, Department of Pediatrics, University of Washington Seattle United States; 8 Public Health Research Institute and Department of Medicine, Rutgers New Jersey Medical School Newark United States; 9 Department of Biochemistry and Molecular Genetics, University of Illinois, College of Medicine Chicago United States; DaVita Labs United States; Icahn School of Medicine at Mount Sinai United States

**Keywords:** COVID-19, PASC, immunopathology, innate immunity, adaptive immunity

## Abstract

With a global tally of more than 500 million cases of severe acute respiratory syndrome coronavirus 2 (SARS-CoV-2) infections to date, there are growing concerns about the post-acute sequelae of SARS-CoV-2 infection (PASC), also known as long COVID. Recent studies suggest that exaggerated immune responses are key determinants of the severity and outcomes of the initial SARS-CoV-2 infection as well as subsequent PASC. The complexity of the innate and adaptive immune responses in the acute and post-acute period requires in-depth mechanistic analyses to identify specific molecular signals as well as specific immune cell populations which promote PASC pathogenesis. In this review, we examine the current literature on mechanisms of immune dysregulation in severe COVID-19 and the limited emerging data on the immunopathology of PASC. While the acute and post-acute phases may share some parallel mechanisms of immunopathology, it is likely that PASC immunopathology is quite distinct and heterogeneous, thus requiring large-scale longitudinal analyses in patients with and without PASC after an acute SARS-CoV-2 infection. By outlining the knowledge gaps in the immunopathology of PASC, we hope to provide avenues for novel research directions that will ultimately lead to precision therapies which restore healthy immune function in PASC patients.

## Introduction

Many adults and children experience prolonged symptoms after severe acute respiratory syndrome coronavirus 2 (SARS-CoV-2) infection. These ongoing, relapsing, or new symptoms as well as other detrimental health effects are referred to as post-acute sequelae of SARS-CoV-2 infection (PASC) or ‘long COVID’ when present 4 or more weeks after the acute phase of SARS-CoV-2 infection. While it has become clear that PASC can present with a myriad of symptoms affecting different organ systems, why this occurs is still largely unknown. Understanding the mechanisms underlying PASC is crucial for the development of appropriate preventive and treatment strategies.

Immune dysregulation has been established as a key pathogenic feature of acute COVID-19 and also plays a role in the pathogenesis of systemic and tissue-specific PASC manifestations. However, the causal mechanisms of immune dysfunction in PASC need to be defined. The mechanisms of immune dysregulation likely vary depending on the PASC clinical phenotype, the severity of the initial COVID-19 infection, as well as the specific tissues involved. Well-designed studies can help elucidate the role of immune mechanisms in PASC.

The NIH-funded Researching COVID to Enhance Recovery (RECOVER) Initiative, which was launched in February 2021, involves more than 100 researchers across the United States who are carrying out studies on PASC (also commonly referred to as ‘long COVID’), either via new studies or by leveraging pre-pandemic long-term cohort studies, implementing the same set of protocols. A central part of RECOVER is the generation of a large SARS-CoV-2 recovery cohort where researchers will collect data from individuals across the lifespan using electronic health records and patient-completed surveys. A subset of participants will also provide biospecimens including blood, nasal swabs, saliva, lymph node aspirants, etc. (https://recovercovid.org/protocols). The ultimate goal is to use the data and resource products to develop preventive and therapeutic treatments in light of the incalculable public health impact of COVID-19 and PASC.

The Mechanistic Pathways Committee within the RECOVER consortium focuses on three mechanistic priority areas – immunopathology, viral persistence, and tissue damage – as the potential salient mechanisms underlying various clinical PASC phenotypes. This perspective article (alongside the companion papers on viral persistence [Chen et al., 2023] and PASC tissue damage [Sherif et al., 2023]) aims to provide a brief overview of current knowledge and raise important questions and hypotheses in these three areas to identify gaps in understanding the mechanisms of disease severity and sequelae of COVID-19, generate new thoughts and ideas, that will help design studies and direct research efforts.

In this article, multiple researchers with wide-ranging expertise in adult and pediatric medicine came together to review the role of the innate and adaptive immune systems in the pathogenesis of acute SARS-CoV-2 infection and PASC. The focus on immune mechanisms here emphasizes the dysregulation of immune processes that are intertwined with the viral persistence and tissue-specific pathologies [discussed in companion reviews] that will likely be needed to address the range of different PASC subtypes which have been defined recently by RECOVER investigators (Reese et al., 2023). Potential hypotheses for PASC-related immune dysregulation are also discussed with suggestions for studies that can help answer these vital questions. Even though there are likely key differences between immune mechanisms during acute COVID-19 and those involved in PASC, the acute immunopathology of COVID-19 can serve as a starting point for examining candidate pathways involved in the immunopathology of PASC. We will therefore outline the established knowledge on acute COVID-19 as well as the limited data on the immunopathology of chronic PASC to highlight important new avenues for research on PASC immunopathology.

### The role of the innate immune system in COVID-19 and PASC

#### Innate immunity

The severe acute respiratory distress syndrome (SARS) caused by the SARS-CoV-2 infection is thought to be driven by an exuberant and dysregulated innate immune response, that is coupled with poor adaptive immune responses (Lowery et al., 2021; Diamond and Kanneganti, 2022). The sensing of initial infection triggers a type I interferon response, and the magnitude of this early response can suppress infection effectively in a majority of subjects. However, when type I responses are delayed (Galani et al., 2021), or impaired due to autoimmune neutralizing responses (Bastard et al., 2020), severe disease has been found to occur. During acute infection, the viral RNA, dsRNA replication intermediates are recognized by pattern recognition receptors, TLR3, TLR7, RIG-I, and MDA5, and in order to replicate the virus antagonizes these responses through the activity of a number of virally encoded genes, including no fewer than 13 genes: Nsps 1, 3, 12, 13, 14, 15, ORFs 3a, 3b, 6, 8, 9b, M, and S (Xia and Shi, 2020; Beyer and Forero, 2022). Subsequent dysregulation of proinflammatory cytokines including IL-6, IL-8, IL-10, and tumor necrosis factor (TNF) along with elevated chemokines ensues in severe disease (Blanco-Melo et al., 2020; Huang et al., 2020; Zhou et al., 2020b; Merad et al., 2021). With severe disease, many patients suffer from prolonged hospitalizations associated with prolonged elevated cytokines. At present the extent to which elevated cytokines are characteristic of PASC is still somewhat unclear. The presence of viral RNA in plasma has been described as an early factor that anticipates PASC sequelae (Su et al., 2022). The persistence of virus in sites that are not routinely sampled or in immunological sanctuary sites such as the gastrointestinal tract, olfactory system, or the brain, may cause local immune activation, and inflammation that drive symptoms of PASC (Gaebler et al., 2021; Klein et al., 2022), though the extent to which this persistence or associated tissue injury causes PASC, and if it is mechanistically tied to symptoms is unclear; this topic is discussed in more detail in the companion articles on viral persistence (Chen et al., 2023) and tissue injury (Sherif et al., 2023).

#### Neutrophils and NETs in acute COVID-19

Several studies have suggested that excessive neutrophil activation is a hallmark of severe acute COVID-19. The degree of neutrophil activation as early as the first week following the diagnosis of SARS-CoV-2 infection served as an important predictor of whether the disease trajectory would take a more severe course (Aschenbrenner et al., 2021; Meizlish et al., 2021; Vanderbeke et al., 2021; Wang et al., 2022). Therapeutic approaches to modulate neutrophil activation and defined neutrophil subpopulations have suggested that neutrophil activation is not just a biomarker of severe disease but instead plays a key pathogenic role in the progression to severe COVID-19 (Hoang et al., 2021; Sinha et al., 2022). Underlying mechanisms could include the amplification of inflammatory and pro-thrombotic loops via interactions with other immune cells that result in the formation of cytokine storms (Kaiser et al., 2021; Vanderbeke et al., 2021).

Formation of NETs (neutrophil extracellular traps) emerged as another key mechanism by which neutrophils could promote severe COVID-19. NETs are extracellular chromatin filaments which are released by neutrophils and are typically coated with cytosolic proteins such as calprotectin and granular proteins such as myeloperoxidase or neutrophil elastase (Henriksson and Kelter, 1987; Jorch and Kubes, 2017). They are thought to serve a host defense function by trapping pathogens, but several studies have documented proinflammatory and thrombogenic roles of NETs in a wide range of acute and chronic diseases (Henriksson and Kelter, 1987; Jorch and Kubes, 2017). Such maladaptive roles of NET formation have also been observed in several respiratory diseases such as acute lung injury, COPD, or asthma where NETs are both cytotoxic to the lung epithelium and endothelium while concomitantly amplifying inflammatory cascades, activating platelets, and inducing autoimmunity (Twaddell et al., 2019). Several studies examined autopsies of patients with severe COVID-19 and found excessive formation of NETs in the lung tissue (Middleton et al., 2020; Radermecker et al., 2020; Veras et al., 2020). Additionally, blood samples from patients with severe COVID-19 showed significantly higher circulating NETs (Veras et al., 2020; Zuo et al., 2020; Carmona-Rivera et al., 2022), as well as the greater propensity of circulating neutrophils from severe COVID-19 patients to form NETs ex vivo (Skendros et al., 2020). As with overall neutrophil activation, detection of NETs can provide important prognostic information for risk stratifying patients (Huckriede et al., 2021; Ng et al., 2021; Carmona-Rivera et al., 2022).

Although NETs are elevated in acute COVID-19 and the levels correlate with disease severity (Veras et al., 2020; Huckriede et al., 2021), their complexity (net-like structures composed of DNA–histone complexes and proteins released by activated neutrophils) makes it challenging to directly infer underlying mechanisms to explain the observed pathobiology. Multiple studies have quantified NETs with distinct assays that vary in terms of specificity, sensitivity, and objectivity. Each assay analyzes specific components such as DNA release in supernatants of stimulated leucocytes, DNA-complexed with myeloperoxidase or neutrophil elastase, presence of citrullinated histones by fluorescence microscopy or flow cytometric detection of NET components. Optimization of assays for quantification of NETs will further improve mechanistic studies investigating which components of NETs are driving the pathobiology. It is unclear which aspects of NETs lead certain patients infected with SARS-CoV-2 to develop a more severe phenotype.

#### Neutrophils and NETs in PASC

While these above-mentioned studies have established the critical role of excessive neutrophil activation and NET formation in severe acute COVID-19, little is known about the potential role of neutrophils and NETs on long COVID or PASC. Studies in the subacute period following the acute SARS-CoV-2 infection suggest that persistence of neutrophil activation is a key determinant of disease severity (Frishberg et al., 2022; Wang et al., 2022). Neutrophil responses also appear to remain abnormal for several months after the acute SARS-CoV-2 infection has subsided (Jukema et al., 2022). However, this raises the intriguing question of how short-lived neutrophils (24–72 hr) can ‘remember’ an acute SARS-CoV-2 infection that resolved weeks or months earlier. NETs are typically formed in the acute infection period but if the propensity to form NETs is increased in PASC, then how would such persistent programming occur? One possibility is that long-lived parenchymal, vascular, and immune cells in the tissue have been reprogrammed by the preceding SARS-CoV-2 infection for example by epigenetic shifts, and instruct incoming neutrophils (which themselves can be short-lived cells) to take on pro-activation and NET-forming signatures. Another possibility would be that the bone marrow environment has been reprogrammed by the preceding SARS-CoV-2 infection which in turn impacts the generation and differentiation of myeloid progenitors so that newly differentiated neutrophils may be more prone to excessive activation. Key questions regarding the roles of neutrophils and NET formation in PASC include: (1) is there evidence of persistent chronic neutrophil activation and an increased spontaneous NET formation in PASC? (2) Is there evidence of augmented propensity for excessive neutrophil activation and NET formation when PASC patients encounter de novo infectious and non-infectious stimuli (second-hit scenario)? (3) Are changes in PASC neutrophil phenotype tissue specific or systemic and can they be traced back to the neutrophil differentiation or maturation in the bone marrow? (4) Can multi-omic analyses help characterize distinct pathogenic neutrophil subpopulations in PASC in order to identify the underlying molecular mediators? (5) Does NET release in PASC promote autoantibody generation and exacerbation of autoimmunity in PASC?

#### Monocytes and macrophages in COVID-19

Monocytes and macrophages are myeloid phagocytic cells that are sentinel responders to infection including SARS-CoV-2. Both monocytes, which circulate in the periphery and are recruited to affected tissues, and macrophages, which exist in tissue and can be either monocyte derived or tissue resident, sense invasive infection via antibody-independent and -dependent pathways. Activation by pathogen-associated molecular patterns or damage-associated molecular patterns in these cells leads to inflammasome activation, triggering inflammatory cytokine release and pyroptosis, or proinflammatory cell death (Swanson et al., 2019; Vora et al., 2021; Junqueira et al., 2022). While the monocyte and macrophage response can be helpful with viral clearance after infection, an exaggerated or dysregulated immune response can exacerbate tissue damage and delay recovery (Schulert and Grom, 2015; Merad and Martin, 2020; Karki et al., 2021; Merad et al., 2022).

Indeed, a hyperactivated and dysregulated monocyte/macrophage inflammatory response to SARS-CoV-2 is thought to be a major contributor to disease severity and death in patients with COVID-19. Excess circulating immature monocytes, inflammatory monocytes, and myeloid progenitors have been found to be pathognomonic of severe disease (Silvin et al., 2020; Zhou et al., 2020a; Merad et al., 2022). A recent study reported that 6% of blood monocytes in patients with COVID-19 were infected with SARS-CoV-2, and that this infection was antibody dependent (Junqueira et al., 2022). Infected cells underwent inflammasome-mediated pyroptosis, likely leading to systemic inflammation and COVID-19 pathogenesis. Tissue macrophages have also been shown to be increased in both bronchoalveolar fluid and lung tissue (Liao et al., 2020; Song et al., 2020), as well as show signs of inflammasome activation (Junqueira et al., 2022). It has been demonstrated in a humanized mouse model that SARS-CoV-2 can infect and replicate in human macrophages and these infected macrophages have an inflammatory phenotype characterized by inflammasome activation and enrichment in the expression of several cytokines (Sefik et al., 2022). A recent study reported that infection of adipose tissue-resident macrophages and resulting inflammation may also potentially be a mechanistic explanation of the epidemiologic association between obesity and COVID-19 disease severity (Martínez-Colón et al., 2022).

In the setting of severe SARS-CoV-2, monocytes and macrophages produce excessive amounts of inflammatory molecules that promote vascular permeability and organ damage, including IL-1α, IL-1β, IL-6, IL-7, TNF, type I and II IFN, and the inflammatory chemokines CCL2, CCL3, and CXCL10 (Merad and Martin, 2020). This cytokine profile resembles those seen in cytokine release syndromes such as macrophage activation syndrome suggesting that dysregulated monocyte/macrophage responses contribute to COVID-19-related hyperinflammation and disease severity (Schulert and Grom, 2015; Mehta et al., 2020). The high levels of IL-6 seen in sera of patients with severe disease led to identification of therapeutics inhibiting IL-6 which have been used successfully in patients with moderate-to-severe COVID-19 infection. Medications used include anti-IL-6 receptor monoclonal antibodies (e.g., tocilizumab) and anti-IL-6 monoclonal antibodies (i.e., siltuximab). JAK/STAT pathway blockers have also been used in dampening IL-6 signal transduction (Aliyu et al., 2022). Granulocyte–macrophage colony-stimulating factor (GM-CSF) levels are also elevated in patients with severe disease and therapies targeting GM-CSF have shown promise in reducing inflammation and improving outcomes (Lang et al., 2020).

#### Monocytes and macrophages in PASC

There is limited research on the role of monocytes and macrophages in PASC. Aberrant activation of monocytes and macrophages can contribute to both the hyperinflammation and the coagulation abnormalities that may lead to progressive tissue damage (Siddiqi and Mehra, 2020). One study found that patients with PASC had higher frequencies of CD14+ CD16+ intermediate monocytes, and activated CD38+ HLADR+ myeloid cells, up to 8 months after the initial mild-to-moderate SARS-CoV-2 infection, in comparison with SARS-CoV-2 unexposed controls (Phetsouphanh et al., 2022). Another study also found that patients with PASC had significantly elevated levels of both intermediate (CD14+, CD16+) and non-classical (CD14−, CD16+) monocytes up to 15 months post-infection (Patterson et al., 2021). Although the lifespan of circulating monocytes is on the order of days, imprinting of hematopoietic progenitor cells in the bone marrow, and/or T cell/monocyte interactions, might explain these long-lived changes in the circulating monocyte compartment.

Persistent or abnormal activation of proinflammatory pathways in tissue-specific macrophages could also have a role in the plethora of PASC symptoms involving multiple organ systems that is seen in these patients. Macrophages are present in multiple tissues that include brain microglia, liver Kupffer cells, lung alveolar and interstitial macrophages, and adipose tissue, and play important roles in immune defense and tissue homeostasis (Bassler et al., 2019). SARS-CoV-2 infection has been shown to lead to an inflammatory transcriptional and metabolic imprint in the macrophage compartment that lasts for at least 5 months. Monocyte-derived macrophages from convalescent SARS-CoV-2-infected individuals showed a downregulation of pro-resolving factors and an increased production of proinflammatory eicosanoids (Bohnacker et al., 2022). To which degree persistent infection or activation of tissue macrophages is a driver of continued inflammation and PASC remains unclear.

Peripheral immunophenotyping in children with Multisystem inflammatory syndrome in children (MIS-C) has shown that monocytes are one of the immune cell populations that are activated during the acute phase, although the classical monocyte proportions were unchanged (Carter et al., 2020). Another study found that although classical monocyte cell frequencies in patients with MIS-C, COVID-19, and healthy controls were similar, classical monocytes from patients with MIS-C showed repressed inflammatory signatures compared to the other two groups. Interestingly, they also found that the lymphocytes and dendritic cell populations tended to have lower inflammatory signatures instead in the acutely infected and previously health controls (Sacco et al., 2022). The differential immunological profile among the monocytes, lymphocytes, and dendritic cells may therefore have a role in development of sequelae after SARS-CoV-2 infection (Knoll et al., 2021).

Altogether, monocyte- and macrophage-driven hyperinflammation and coagulopathy both contribute to disease severity in patients with COVID-19 and could also have a significant role in development of sequelae including MIS-C and PASC (Knoll et al., 2021). A better understanding of the molecular mechanisms that lead to these various responses could lead to better therapeutic and preventive strategies. As tissue-resident macrophages typically have a longer lifespan than monocytes and because macrophages exhibit significant differences in terms of phenotype depending on where they reside, it will be important to examine the tissue-specific dynamics of monocytes and macrophages. Unbiased approaches such as transcriptomics and proteomics of distinct monocyte and macrophage populations will allow for the identification of novel pathogenic mediators in PASC.

#### Mast cells in COVID-19 and PASC

Mast cells are innate immune cells found primarily near tissue–environment interfaces, such as skin and mucosa. They possess cell-surface receptors including the high-affinity IgE receptor Fc which react to various triggers and release many proinflammatory mediators including histamine, heparin, cytokines, prostaglandins, leukotrienes, and proteases. Mast cell activation syndrome is characterized by a massive and unregulated release of these mediators, which produces often severe inflammatory and allergic symptoms such as anaphylaxis (Valent et al., 2012; Frieri, 2018). Accordingly, mast cells have been theoretically linked to the cytokine storm associated with acute SARS-CoV-2 infection, and some studies have shown evidence for mast cell activation in lungs and sera of acute COVID-19 patients. At least one autopsy study showed increased mast cell accumulation and activation in lungs of COVID-19 patients compared with controls without lung pathology (Budnevsky et al., 2022). It has been proposed that mast cells play a role in the pathogenesis of PASC, but data demonstrating such causal roles are limited (Afrin et al., 2020; Davis et al., 2023). Some studies have shown that many PASC patients show symptoms which emulate MCAS (mast cell activation syndrome) (Weinstock et al., 2021). Wechsler et al., 2022 assessed levels of mast cell-derived proteases to evaluate mast cell activation in symptomatic PASC patients. Active tryptase levels were significantly elevated in PASC sera compared to asymptomatic post-COVID patients and heathy controls. Serum tryptase levels also correlated with IL-6 levels in the PASC patients. The importance for PASC patients relates to current pharmacological strategies such as antihistamines to treat MCAS, and their potential application in PASC (Davis et al., 2023). However, additional research is required to prove that mast cells substantially contribute to PASC symptomatology and that such therapeutic approaches would be effective.

### The role of the adaptive immune system in COVID-19 and PASC

#### T and B cells in COVID-19 and PASC

The role of the adaptive immune compartment in the pathophysiology of PASC is an active area of investigation. The SARS-CoV-2-specific T cell response has been well characterized in acute and convalescent COVID-19 (Sette and Crotty, 2021; Moss, 2022), and studies suggest these responses likely contribute to virus clearance (Tan et al., 2021), correlate with both peak and durable antibody responses (Rydyznski Moderbacher et al., 2020; Cohen et al., 2021; Dan et al., 2021; Zuo et al., 2021a; van der Ploeg et al., 2022), and may help protect against re-infection (Moss, 2022). Although the presence of SARS-CoV-2-specific T cell responses has been associated with milder disease in acute and convalescent COVID-19 patients (Rydyznski Moderbacher et al., 2020; Sekine et al., 2020), a robust, proinflammatory T cell response has also been associated with more severe manifestations of acute COVID-19 (Popescu et al., 2022; van der Ploeg et al., 2022). Similarly, a dysregulated B cell response, including germinal center derangements and extrafollicular B cell activation, has also been implicated in the pathogenesis of severe and critical COVID-19 (Kaneko et al., 2020; Nielsen et al., 2020; Woodruff et al., 2020; Röltgen et al., 2022).

Several studies have evaluated the role of SARS-CoV-2-specific T cells in the pathophysiology of PASC, with somewhat mixed findings. An earlier study identified lower and more rapidly waning nucleocapsid-specific cytotoxic CD8+ T cells among individuals with PASC compared with those without PASC (Peluso et al., 2021a). Similarly, another study found that patients with PASC had reduced CD4+ and CD8+ effector memory cell numbers and increased PD-1 (programmed cell death protein 1) expression on central memory cells (Glynne et al., 2022). However, these studies could not determine whether this represented decreased functional capacity of virus-specific CD8+ T cells vs. generalized dysfunction/persistent antigenic stimulation. In contrast, a more recent study reported that patients with pulmonary PASC had significantly higher frequencies of interferon-gamma (IFNg) and TNF producing SARS-CoV-2-specific T cells in peripheral blood compared to individuals without PASC (Littlefield et al., 2022). This study also observed that higher levels of immune activation – manifested by circulating plasma inflammatory biomarkers (IL-6 and CRP) – positively correlated with both PASC and the frequency of SARS-CoV-2-specific T cells (Littlefield et al., 2022). In line with these observations, studies have reported that patients with pulmonary manifestations of PASC have dysregulated tissue-resident memory (TRM) CD8+ T cell responses in bronchial airway lavage fluid, and that these findings were associated with impaired lung function (Vijayakumar et al., 2022). Persistence of specific TRMs has been previously noted after other viral infections and may be related to continued viral antigen presence. However, the limited number of PASC studies has not tested specific TRM responses to SARS-CoV-2 antigens.

Few studies have examined longitudinal changes in T cell profiles in patients with PASC. A few studies have documented T cell dynamics at multiple time points after COVID-19 infection in peripheral blood (Wiech et al., 2022). These studies have found that recovery from COVID-19 leads to a functional shift in T cell subsets after the ﬁrst 3 months post-infection, and that these longitudinal T cell dynamics are associated with the severity of SARS-CoV-2 infection. Whether these longitudinal changes play a role in the pathophysiology of PASC remains an active area of investigation.

Regarding the role of B cells in PASC, several studies have detailed the presence of B cell-derived autoantibodies in the pathogenesis of PASC (discussed in further detail below). More recently, a transient, naive-derived antibody-secreting B cell (ASC) compartment was identified that was enriched in autoreactive potential. These cells were found to emerge during the acute phase of severe COVID-19 and regress gradually during recovery in most, but not all, patients (Woodruff et al., 2022). Importantly, autoantibodies persisted in a subset of patients with post-acute sequalae, suggesting that these ASC induced during acute COVID-19 may indeed play a role in the pathogenesis of PASC, although this remains to be determined.

Importantly, systems-based analyses have also implicated broad adaptive immune activation in the pathophysiology of PASC (Peluso et al., 2021b; Su et al., 2022). In a large, multi-omics analysis of patients with and without PASC, gastrointestinal PASC was correlated with expansion of both SARS-CoV-2- and CMV-specific populations of cytotoxic CD8+ and CD4+ T cell populations (Su et al., 2022). Notably, these populations were activated not during acute disease, but during convalescence, at the time PASC was identified (Su et al., 2022). This study also implicated SARS-CoV-2 RNAemia and Epstein–Barr virus (EBV) viremia as PASC-anticipating factors (Su et al., 2022). In another multi-omics study utilizing deep immunophenotyping, PASC was associated with increases of multiple adaptive immune populations, including exhausted T cells, IL-4/IL-6-secreting CD4+ T cells, and activated B cells (Klein et al., 2022). Individuals with PASC in this study had higher SARS-CoV-2-specific IgG against Spike and S1, but also had higher antibody responses against herpesvirus lytic antigens, as compared to vaccination-matched controls (Klein et al., 2022). Altogether, these data suggest that chronic inflammation in individuals with PASC – possibly due to persistent viral replication and/or reactivation of latent herpesviruses – may be responsible for the perturbations in the adaptive immune compartment observed.

MIS-C and Multisystem inflammatory syndrome in adults (MIS-A) are considered PASC syndromes yet exhibit a different time course and presentation than other chronic long-COVID syndromes. Children with MIS-C have significantly lower virus-specific CD4+ and CD8+ T cell responses to major SARS-CoV-2 antigens compared with children convalescing from COVID-19. However, T cell repertoire in MIS-C patients has been found in several studies to skew toward both CD4+ and CD8+ T cells expressing the T cell receptor (TCR) *TRBV11-2* gene, a hallmark of superantigen-mediated activation (Moreews et al., 2021; Porritt et al., 2021; Sacco et al., 2022). Thus, this shift in TCR repertoire supports the theory that portions of the SARS-CoV-2 spike protein function as a superantigen and activates T cells according through these TCRs (Noval Rivas et al., 2022). A more detailed discussion on the immunological changes in MIS-C can be found in the companion paper on pathogenic mechanisms of PASC (Sherif et al., 2023). While shifts toward TRBV genes have been noted in adults with severe acute COVID-19 (Cheng et al., 2020) limited information currently exists for TCR repertoires in adult patients with long COVID. However, in single patient with T cell myocarditis with MIS-A, post-mortem analyses revealed T lymphocytes within the heart expressing various TRBV genes (Vannella et al., 2021).

**Figure 1.**
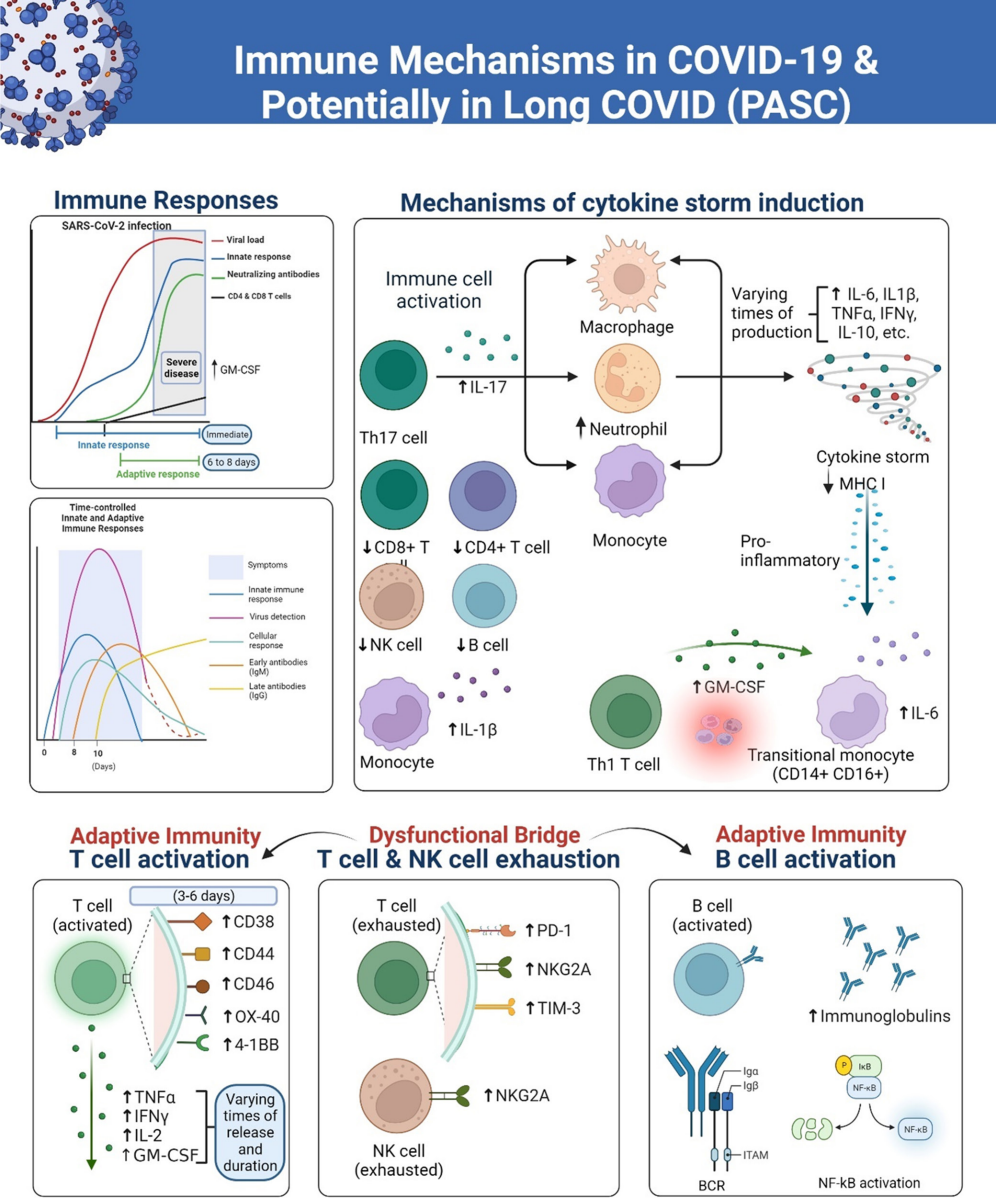
A simplified overview of responses of the immune system to the SARS-CoV-2 virus, over various time courses that lead to severe COVID-19 and long COVID (PASC) in some patients. The innate immune response serves as the initial line of defense against the virus, involving the activation of immune cells such as monocytes, natural killer (NK) cells, dendritic cells, and macrophages. These cells release cytokines and chemokines to recruit other immune cells, such as T cells and neutrophils, to the site of infection. Later in the course of the infection, the adaptive immune cells including B plasma cells release antibodies. However, the overreactive immune responses trigger inflammatory conditions that are sustained in PASC due to dysregulation of the immune system. The diagram was created using the Biorender.com software.

Figure 1 shows the arcs of the immune response in COVID-19 including the theoretical schematics of the kinetics of immune responses to SARS-CoV-2 in severe and fatal COVID-19. The ‘Innate immunity’ chart line in the model refers specifically to the peak blood kinetics of innate cytokines and chemokines, which occur in a localized manner throughout the duration of an infection. In the time-controlled adaptive response schematic sketches in the figure, the time points show the important differences in the presence or absence of T cell responses and the magnitude of the viral load (Sette and Crotty, 2021). Furthermore, corresponding changes in gene expression over time in the generation of primary and secondary immune responsive proteins as the disease progresses have been shown to yield valuable insights into the biological mechanisms that distinguish adaptive from maladaptive events (Wang et al., 2022).

Overall, while GM-CSF and cytokines are not released simultaneously during COVID-19 infection, they are both involved in the immune response to the virus and may play a role in disease severity. When the adaptive immune response is activated, B cells producing antibodies and T cells targeting the virus proliferate. Once the virus is cleared, most immune cells decrease in number and return to a resting state. However, in some individuals like long haulers, a subset of immune cells persists, leading to chronic inflammation and tissue damage (Figure 1). This uncoordinated and dysfunctional adaptive immune response to SARS-CoV-2 infection and viral persistence can result in a range of long-COVID symptoms. There continue to be significant gaps in our knowledge of the clinical spectrum of COVID-19, immune signaling pathways, systemic effects, and long-term pathological signatures and this must be addressed through further investigations into these different immune mechanisms depicted.

#### Autoantibodies in COVID-19 and PASC

Early in the COVID-19 pandemic it was suggested that autoimmunity might have a role in the hyperinflammatory disease and respiratory failure observed in severe and fatal presentations of SARS-CoV-2 infection (Favalli et al., 2020; Zhou et al., 2020b). Indeed, COVID-19 can be associated with hematological abnormalities and system dysfunction (neurological, pulmonary, cardiovascular, gastrointestinal, constitutional) resembling those previously observed in autoimmune diseases or autoimmune manifestations associated with various infectious agents, in particular viruses, mostly of unknown etiology (reviewed in Ehrenfeld et al., 2020). Moreover, in a proportion of cases, severe pulmonary manifestations have been associated with elevated concentrations of autoantibodies neutralizing type I IFNs (Bastard et al., 2020; Combes et al., 2021), a finding that has been replicated in numerous studies (reviewed in Zhang et al., 2022). These results are in keeping with a key event in COVID-19 pathogenesis, that is, dampened or delayed type I IFN responses (Blanco-Melo et al., 2020; Hadjadj et al., 2020; Galani et al., 2021; Kasuga et al., 2021; Lowery et al., 2021), which result in ineffective early control of viral replication, leading to local and systemic inflammation, vasculopathy, and organ damage (Schultze and Aschenbrenner, 2021; Merad et al., 2022).

The full breadth of autoantibodies directed against immunomodulatory proteins goes well beyond targeting type I IFNs. For example, a high-throughput discovery approach interrogating extracellular proteins showed that COVID-19 patients may bear autoantibody reactivities directed against cytokines, chemokines, and cell-surface proteins (Wang et al., 2021). Some of these reactivities may have functional effects, including neutralization of cytokines and chemokines or engagement of FcR-dependent effector functions, such as antibody-dependent cellular phagocytosis, potentially leading to immune cell depletion. Furthermore, excess autoantibodies targeting antigens expressed on the surface of immune cell subsets were associated with reduced frequencies of the corresponding immune cell types, again suggestive of functional links between autoantibodies and selective immune cell depletion (Wang et al., 2021). Thus, autoantibody production may affect various innate and adaptive immune functions and contribute to ineffective or harmful immune responses.

Autoantibodies associated with COVID-19 may be outright pathogenic. For example, antiphospholipid antibodies may activate endothelial cells and platelets and stimulate neutrophils to release NETs (Knight et al., 2021). Increased NET production correlates with thrombosis and COVID-19 severity (Zuo et al., 2021c). Moreover, elevated antibodies binding to NETs, which impair NET degradation and likely activate complement, also correlate with COVID-19 severity (Zuo et al., 2021b). Thus, abnormal coagulation, vasculopathy, and micro- and macrovascular thrombosis, which are characteristic of severe COVID-19 (Perico et al., 2021), may be associated, at least in part, to the production of these autoantibodies (reviewed in Knight et al., 2021). Autoantibody production has also been correlated with severe disease manifestations secondary to SARS-CoV-2 infection in children. In particular, MIS-C which is characterized by fever, rash, and gastrointestinal symptoms and may also have life-threatening cytopenias, coagulopathy, myocardial dysfunction, coronary aneurysms, and shock (Chou et al., 2022) has been associated with the presence of autoantigens characteristic of classical autoimmune diseases (Gruber et al., 2020). Thus, a broad repertoire of autoantibodies is seen during severe clinical manifestations of SARS-CoV-2 infection across all age groups.

Multiple mechanisms likely underlie the production of autoantibodies in patients with severe COVID-19 manifestations. In one scenario, autoantibodies may precede infection with SARS-CoV-2 and be genetically determined, as proposed, for example, for neutralizing autoantibodies against type I IFNs (Bastard et al., 2020) that, as mentioned above, dampen the early innate antiviral responses, leading to uncontrolled viral replication, hyperinflammation, and tissue damage (Merad et al., 2022). However, more recent data show very low prevalence of IFN autoantibodies in PASC patients, indicating that these antibodies may not play a role in the pathogenesis of long COVID (Peluso et al., 2023). This highlights the need to define whether preexisting auto-antibodies may predispose subjects infected with SAS-CoV-2 to develop more severe forms of PASC immunopathology. In genetically predisposed individuals SARS-CoV-2 infection could act as trigger for new-onset rheumatic autoimmune diseases. The various hypothetical mechanisms include bystander activation triggered by the cytokine storm, viral persistence causing polyclonal activation and the formation of NETs (Shah et al., 2020; Winchester et al., 2021; Zacharias et al., 2021). Arthritis and vasculitis are the most common new-onset rheumatic diseases seen in COVID-19 patients (Gracia-Ramos et al., 2021), however they are not very common.

In a second scenario, autoantibody production occurs de novo in SARS-CoV-2 infection, indicating that severe COVID-19 can break tolerance to self. New-onset IgG autoantibodies were found to react to autoantigens associated with classical and rare autoimmune diseases – ranging from antinuclear antibodies (ANA) to antibodies against connective tissue and various secreted proteins, including cytokines, chemokines, and growth factors (Chang et al., 2021). De novo production of autoantibodies implicates extrafollicular B cells, since, unlike germinal center reactions, extrafollicular maturation lacks some tolerance checkpoints that prevent the activation and maturation of autoreactive B cells, as is the case with system lupus erythematosus (Tipton et al., 2015; Jenks et al., 2018). Indeed, extrafollicular B cells, also known as double-negative B cells, were enriched in severe and fatal cases of COVID-19 (Woodruff et al., 2020) and were found to increase over time even in subjects who had experienced COVID-19 not requiring hospitalization (Mishra et al., 2021).

In a third scenario, autoantibodies may arise from reactivation of latent human herpesvirus in tissues around the time of initial infection. For example, mounting evidence exists that EBV may reactivate during acute SARS-CoV-2 infection, and be related to persistent sequelae such as fatigue and various neurocognitive symptoms (Gold et al., 2021; Peluso et al., 2022a). EBV infection has previously been linked with host antigen molecular mimicry, autoreactive immunity, and pathogenic B cell migration and activity that may lead to diseases such as multiple sclerosis (Bjornevik et al., 2022; Lanz et al., 2022; Robinson and Steinman, 2022). The extent to which latent viral reactivation may play in autoimmunity and PASC is a key area of current research.

Links may exist between the occurrence of PASC and autoimmunity. For example, one study shows that high ANA titers were seen in 43.6% of patients at 12 months post-COVID-19 symptom onset, and the frequency of neurocognitive symptoms correlated with ANA titers (Seeßle et al., 2022). However, a follow-up study in a similar sized cohort was not able to corroborate these findings (Peluso et al., 2022b). Several studies have reported an association between PASC and autoantibodies against G-protein-coupled receptors.(Hohberger et al., 2021; Wallukat et al., 2021; Szewczykowski et al., 2022). Autoantibodies have been included among risk factors for the development of PASC (Su et al., 2022) and it has even been postulated that ‘long COVID’ may be an autoimmune disease (Marshall, 2021). However, in another study, no strong association was observed between autoantigen reactivity and symptoms in COVID-19 convalescent patients. Furthermore, autoantibodies against calprotectin, which was the most common autoantibody target in the study, were associated with return to healthy, normal life 8 months post-infection, suggesting a protective – rather than pathogenic – role for these autoantibodies (Moody et al., 2022). Thus, the role of antibodies in PASC pathogenesis requires further investigation.

The key questions that need to be addressed regarding autoantibodies and PASC immunopathology include: (1) Do preexisting autoantibodies impact the incidence and severity of PASC? (2) Which autoantigens generate de novo autoantibodies, and do these persist beyond the acute phase of SARS-CoV-2 infection? (3) How does genetic predisposition to autoimmune disease affect the de novo diagnosis of classifiable autoimmune disease following the acute SARS-CoV-2 infection? (4) Can we identify specific autoantibodies that are mechanistically tied to specific forms of tissue injury? (5) Does virus-induced autoreactivity underlie some of the clinical phenotypes associated with PASC? These and many other questions raised in this article need to be answered to better understand the mechanisms of PASC.

#### PASC in the context of SARS-CoV-2 endemicity

Much of the research conducted on PASC has occurred in the context of SARS-CoV-2 as an emerging pathogen. As the pandemic has progressed, it appears likely that the virus will become endemic worldwide, with constant circulation in the population resulting in cyclical peaks of emergence. It is clear that some SARS-CoV-2 variants (such as the Omicron variant) have considerable resistance to preexisting immune responses generated by previous infections or vaccination. Recent studies have shown that protection against re-infection with a different variant is reduced and wanes quickly, although it appears protection against severe disease is more robust. In the context of PASC, it is unknown whether re-infection with different variants, or re-infection with the same variant after immunity wanes, affects the frequency or severity of PASC. For example, do suboptimal immune responses (such as binding antibodies that do not neutralize infection or T cell responses that are not robust) lead to reduced or enhanced pathogenicity in PASC? Furthermore, an important question moving forward is how regular, cyclical infection of SARS-CoV-2 on a population level enhances or subdues PASC frequency and severity. Future studies on this topic should carefully take into consideration the previous infection and vaccination history of study participants.

### Conclusion

Both the innate and adaptive immune responses play an essential role in protection from SARS-CoV-2 infection but also have the potential to be detrimental to the host when dysregulated. Overactive and persistent immune responses due to viral or host factors will likely impact the severity of the long-term sequelae like MIS-C, MISA, or PASC. Our current understanding of PASC immunopathology is based on a limited number of PASC studies with few patients, short-term follow-up and educated inferences regarding mechanisms based on acute SARS-CoV-2 infections as well as other known chronic inflammatory diseases. Even at this early stage of investigation, it is becoming apparent that PASC represents a complex immunopathology involving excessive activation of the adaptive and innate immune system, and an interplay between distinct immune cells and the affected tissues. One approach to identifying immunological drivers of PASC would be to initially focus on immune cells and processes that are most likely to explain the chronic nature of the disease. This would include long-lived tissue-resident macrophages, adaptive immune cells that mediate immune memory and auto-antibody production as well as long-term changes in the epigenome of tissue cell types which interact with newly recruited immune cells, thus transmitting the ‘immune memory’ of the disease.

Understanding the immunological underpinnings of PASC will be a vital step toward better deciphering the mechanisms of disease and thus developing targeted therapies. A major challenge to understanding the immune dysregulation in PASC or long COVID is the heterogeneity of symptoms as well as the multitude of cell types involved in the complex pathology of PASC. Analysis of large-scale data from large cohort studies such as RECOVER can help address some of these questions and to identify subjects with PASC who might benefit from immune-modulating therapies without compromising host defense.
